# Clinical evaluation of computer-aided design and three-dimensional printing for completely edentulous mandibular custom tray fabrication: assessing clinician satisfaction

**DOI:** 10.3389/fbioe.2025.1556651

**Published:** 2025-05-21

**Authors:** Tian Zhao, Yi Weng, Ning Li, Yiming Gao

**Affiliations:** Stomatology Centre, Ruijin Hospital, School of Stomatology, Shanghai JiaoTong University, Shanghai, China

**Keywords:** CAD/3D printing, custom tray fabrication, Prosthodontist satisfaction, efficiency, completely edentulous jaw

## Abstract

This study evaluates prosthodontists’ satisfaction and efficiency of custom tray fabrication methods in completely edentulous mandibular jaw patients. A digital workflow was established, incorporating 3D scanning for preliminary impressions, CAD for designing border extensions and ensuring uniform 3D space, and 3D printing for tray fabrication. Three methods were compared: CAD/3D printing, light-cured resin, and impression compound trimming. Prosthodontist satisfaction was assessed using a visual analog scale, and manual fabrication time was analyzed. Dislocation tests were performed to evaluate tray retention. The CAD/3D printing method achieved the highest satisfaction across most parameters (*P* < 0.05), the best stability and retention (*P* < 0.05), and significantly reduced manual fabrication time (*P* < 0.05). The light-cured resin method showed moderate performance, while the impression compound trimming method scored lowest. The CAD/3D printing technique enhances satisfaction, precision, and efficiency, demonstrating significant potential for optimizing prosthodontic workflows. Further exploration in broader clinical applications is recommended.

## 1 Introduction

Edentulism refers to the condition of being toothless, commonly caused by periodontal disease, tooth decay, or trauma. Edentulous jaw represents a significant proportion in edentulous patients, especially in elderly adults (Borg-Bartolo et al., 2022). Dental implant treatment is one of the best options for edentulous patients, however, many patients are restricted to the treatment due to insufficient bone volume or quality, systemic health conditions and high treatment costs (Sailer et al., 2022; Srinivasan et al., 2023). Therefore, conventional complete denture treatment remains the mainstream treatment for edentulism (Goldstein et al., 2021). The impression and cast form the foundation for complete denture fabrication, optimal denture retention and support can only be achieved when impressions are precise and casts are accurately constructed (Kihara et al., 2020; Carneiro Pereira et al., 2021). An optimal edentulous impression must capture precise tissue anatomy, appropriate border extensions, and adequate functional morphology of the surrounding tissues (Koch et al., 2022; Goel et al., 2018). Achieving these objectives depends on the use of custom trays. Custom trays constructed with light-cured resin on preliminary casts can yield highly accurate edentulous impressions; however, the fabrication process is complex and labour-intensive. Therefore, it is crucial to develop custom tray fabrication techniques that are both precise and efficient. Clinicians have been exploring alternative methods for custom tray fabrication. Traditional methods include impression compound trimming and light-cured resin techniques. The impression compound trimming is a simple technique and requires fewer clinical visits, making it suitable for patients with limited mouth opening. However, it often sacrifices accuracy and consistency (Krishna Ch et al., 2013; Yang et al., 2022). The light-cured resin technique offers better accuracy than impression compound trimming, with advantages like fast curing, precise operation, and durable aesthetics in oral prosthetics. However, it is limited by shallow curing depth, high operational demands (Gupta et al., 2017; Negahdari et al., 2022). These limitations highlight the need for innovative approaches that balance precision, efficiency, and clinical practicality.

With the development of digital technologies, digital impression systems have provided a better present and future for the field of dentistry (Ting-Shu and Jian, 2015; Revilla-León et al., 2023a). Digital impression technologies for fixed prosthodontics have significantly reduced chairside time and improve treatment effectiveness, making them widely adopted tools in clinical practice (Papaspyridakos et al., 2023). However, for edentulous patients without clear curvature variations on the alveolar crest surface, direct optical impression techniques (intraoral scanning) cannot accurately capture the three-dimensional (3D) morphology of soft tissues during functional movements and cannot provide morphological information for a functional mould (Revilla-León et al., 2023b). The mandibular edentulous jaw presents challenges in prosthetic dentistry due to its unique anatomical characteristics and biomechanical considerations. Unlike the maxilla, the mandible typically offers less surface area for denture support and exhibits more pronounced resorption patterns over time, which directly impacts denture stability and retention (Caggiano et al., 2023). The dynamic nature of the mandible during functional movements, including lateral excursions, protrusive movements, and varying degrees of opening and closing, presents significant challenges for impression-making procedures (Anitua et al., 2022). Additionally, the influence of the floor of the mouth and tongue actions, such as swallowing and speech patterns, further complicates the process. These complex biomechanical interactions lead to tissue displacement and create border molding requirements that differ substantially from those of the maxilla (Bedrossian and Bedrossian, 2019). Therefore, the current direct digital intraoral impression techniques cannot fully satisfy the need for edentulous mandibular jaws.

It has been reported that custom trays fabricated using computer-aided design (CAD) and 3D printing (3DP) have been used in maxillectomy patients to yield satisfactory prostheses (Deng et al., 2022; Kihara et al., 2021). However, due to the complexity of treating edentulous patients, there is a limited number of studies comparing different methods for the digital design and fabrication of dentures.

This study investigated a digital custom tray fabrication method using 3D scanning for preliminary impression data acquisition, CAD technology for designing border extensions and uniform 3D space, and 3D printing for tray production. Prosthodontist satisfaction and fabrication time were evaluated to compare this method with conventional techniques, aiming to develop an accurate, time-efficient, and labour-saving approach for custom tray fabrication for complete dentures.

## 2 Materials and methods

### 2.1 Study subjects

Thirty-two edentulous mandibular patients (18 men and 14 women; age, 58–84 years; average age, 68.43 ± 3.29 years) who visited the Stomatology Centre at Ruijin Hospital between August 2023 and June 2024 were selected. The inclusion criteria for this study were as follows: Class Ⅲ completely edentulous jaws with (based on Atwood’s classification (Atwood and Coy, 1971)); stable alveolar ridge with no significant resorption or deformities; healthy mucosal tissue without pathological conditions such as ulcers or severe inflammation; no significant mandibular asymmetry or jawbone deformities; be able to cooperate with digital scanning procedures. The exclusion criteria for this study included patients with severe systemic health conditions and neurological or motor disorders; tooth extraction within the last 3 m. All patients were required to understand the study’s objectives and provide informed consent. Approval was sought and obtained from the Ruijin hospital Ethics Committee (Ethical approval number: 2,023,314).

Three doctors with similar professional titles who had worked in the field of clinical prosthodontics for a similar number of years and had never used CAD and 3DP techniques for custom tray fabrication were also recruited. The three doctors were randomly assigned 11 edentulous patients. Custom mandibular trays were fabricated for each patient using the conventional impression compound trimming technique, light-cured resin technique, and CAD and 3DP technique, each of which was implemented at 1-week intervals. Three final mandibular impressions were recorded using these custom trays for each patient.

### 2.2 Custom tray fabrication techniques

**FIGURE 1:**
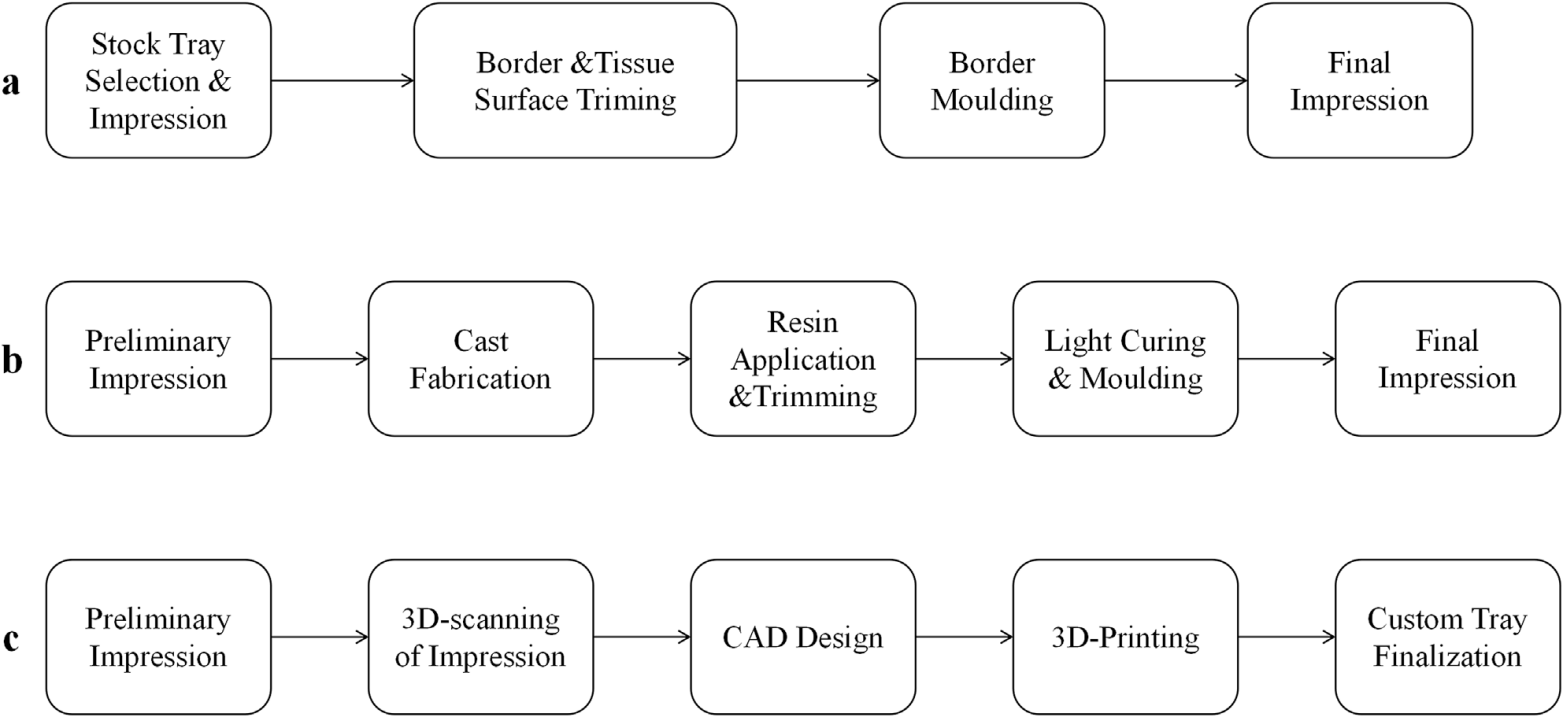
Three methods of custom tray fabrication workflows for edentulous mandibles. **(a)** Impression compound trimming technique; **(b)** Light-Cured Resin Technique; **(c)** CAD/3D printing technique.

Three distinct methodologies for custom tray fabrication were implemented in this study, as illustrated in Figure 1.

**FIGURE 2:**
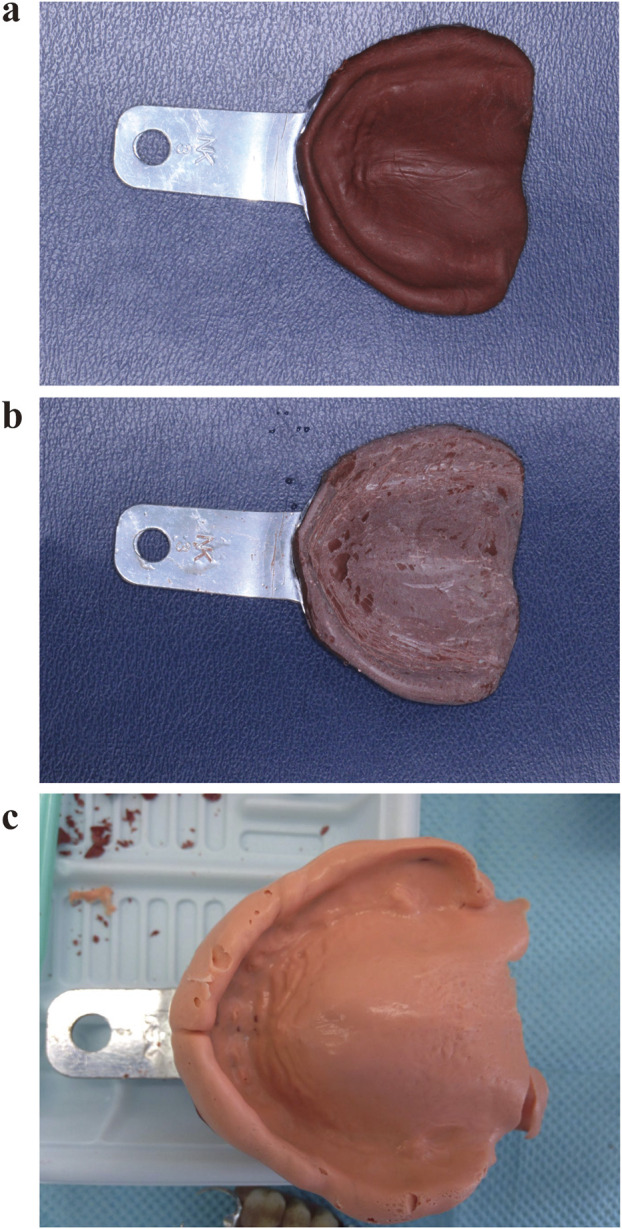
Tray fabrication using the impression compound trimming technique. **(a)** Preliminary impression recorded using impression compound; **(b)** Trimming of the mandibular impression; **(c)**. Final impression recorded using the fabricated tray.

Impression compound trimming technique: A suitable readymade tray was selected and coated with an appropriate amount of softened impression compound to record the preliminary impression (Figure 2a). Then, 2 mm of the paste was trimmed evenly from the edges and tissue surface of the preliminary impression, the undercuts were eliminated, and the buffer area was trimmed to create a custom tray, which was loaded with polyether (3 M Oral Care, United States) to record the final impression (Figures 2b,c).

**FIGURE 3:**
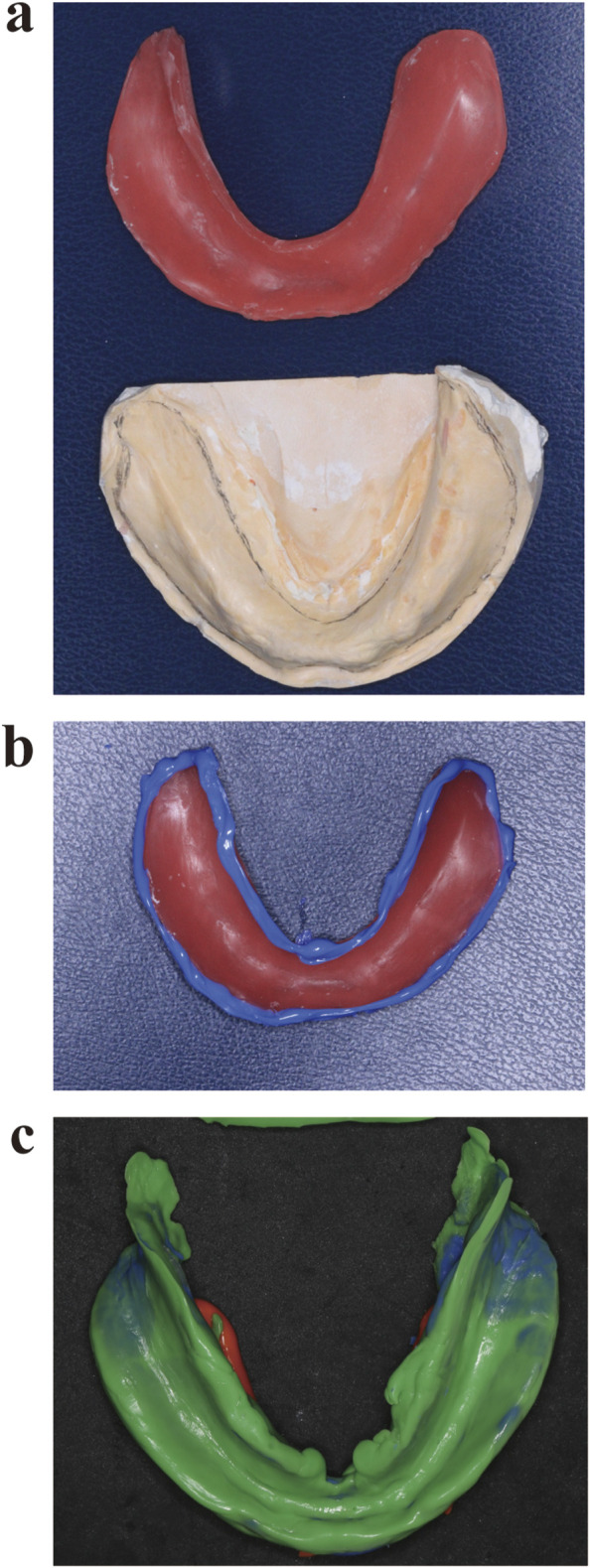
Tray fabrication using the light-cured resin technique. **(a)** Custom tray fabrication using light-cured resin on a preliminary cast fabricated using the impression compound trimming technique. **(b)** Border moulding; **(c)** Final impression recorded using the fabricated tray loaded with polyether.

Light-cured resin (Luxatray^®^, DMG, Germany) technique: After applying the impression compound trimming technique to record the preliminary impression, a preliminary cast was fabricated and the undercuts were buffered and filled (Figure 3a). Then, after treatment with a separating agent, the cast was coated with an approximately 2-mm-thick layer of light-cured resin and trimmed to the appropriate extension range. Custom trays were obtained after light curing, and a special impression compound was used for border moulding (Figure 3b). Light curing was completed and the tray was loaded with polyether to record the final impression (Figure 3c).

**FIGURE 4:**
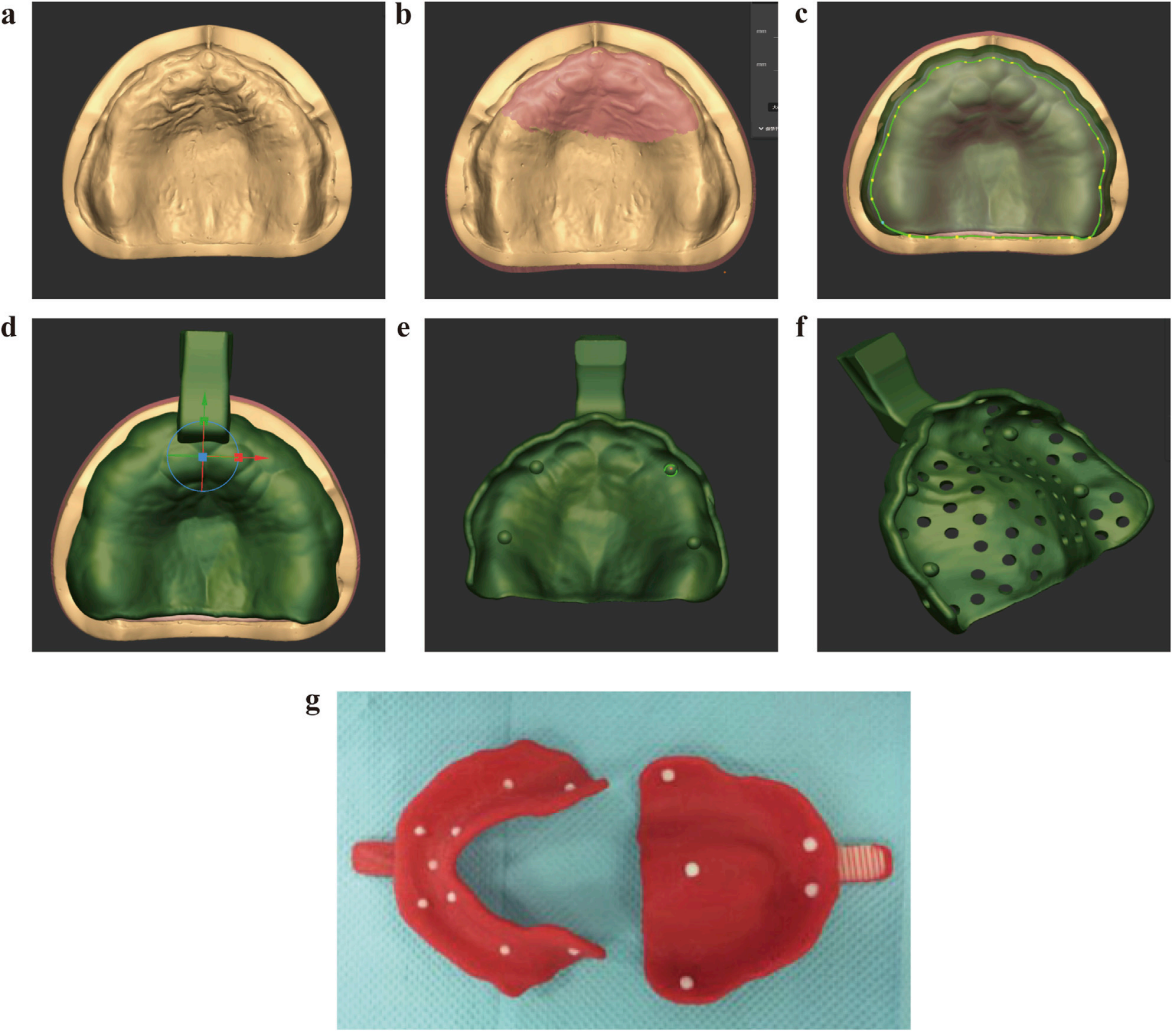
Tray fabrication using the CAD and 3DP technique. **(a)** Preliminary impression with stock tray; **(b)** Scan of the preliminary impression trimmed from paste; **(c)** Virtual spacer design; **(d)** Tray body modeling; **(e)** Positioning hemispheres; **(f)** Final CAD model integration; **(g)** 3D printing.

CAD and 3DP technique: A suitable readymade tray was chosen and loaded with an appropriate amount of softened impression compound to record the preliminary impression. Then, a 3D scanner (Activity880, SmartOptics, Germany) was used to obtain data pertaining to the tissue surfaces by scanning the entire dental arch on the preliminary impression (Figure 4a). The data were saved in the stereolithographic (STL) format and imported into Geomagic 2013 (Raindrop, United States). The mandibular mucosal folds were extracted as margins, which were set as the borders that intercepted with point cloud data on the edentulous mandibular tissue surface on the preliminary impression with the redundant data eliminated (Figure 4b). A suitable path of insertion was chosen and the buccal anterior undercuts interfering with this path were circled, virtually eliminated, and filled. The mandibular torus region was selected and displaced along the normal by 1 mm to complete the virtual buffer; the curved surface boundary was maintained and evenly enlarged along the normal by 2 mm to form the inner surface of the tray, thus reserving an even and consistent 3D space for the impression material (Figure 4c). The thickness of the inner surface of the virtual tray was increased by 2 mm throughout to form the main body of the tray (Figure 4d). Then, in the vertical, anterior-posterior, and left-right planar dimensions of the primary and secondary stress-bearing areas, small hemispheres with a radius of 1.5 mm were designed to facilitate accurate positioning of the fabricated tray; these hemispheres separately coincided with the vertical, anterior–posterior, and left–right insertion directions when the tray was positioned in the oral cavity (Figure 4e). Then, the 3D shape of the handle was fused with the border of the virtual tray to obtain data on the complete shape of the tray (Figure 4f). These data were saved in the STL format and imported into the Replicator 2X 3D printer (MakerBot, United States, layer resolution 0.1–0.3 mm) support software (Imageware 11.0, EDS Corporation, United States) for slicing, following which the custom tray was fabricated from polylactic acid (Ingeo™ Biopolymer 3D860, NatureWorks, United States) using the 3D printer (Figure 4g).

### 2.3 Satisfaction evaluation

Once the final impressions were obtained with all techniques, the satisfaction levels of the attending doctors were evaluated using a designed questionnaire including six questions (Table 1). The doctors provided scores for each item using visual analogy scale (VAS), starting from the point of origin on a straight line. A score of “0” and “100” indicated extreme dissatisfaction and extreme satisfaction, respectively. The higher score indicated better satisfaction level. The degree of satisfaction was quantified based on the linear distance from the origin point, categorized as dissatisfaction (0–40 mm), neutral (41–60 mm), or satisfaction (61–100 mm).

**TABLE 1:** Questions in the questionnaire.

Number	Details
Question 1	Satisfaction levels before intraoral trial of the tray (overall size, shape, grip, *etc.*)
Question 2	Satisfaction levels with border positions during the trial (border ​​extensions, frenal notches)
Question 3	Satisfaction levels with the ease of maintaining tray stability during functional moulding operations
Question 4	Satisfaction levels with the recorded final impression (completeness, border morphology, impression material thickness)
Question 5	Satisfaction levels with the difficulty in achieving a good-quality final impression (repetitions, efficiency)
Question 6	Overall satisfaction level

### 2.4 Manual fabrication time record

The manual fabrication times for both the light-cured resin and CAD/3D-printed trays were documented using digital chronometers (Seiko S054, Japan). The workflow was segmented into three main phases for comparative analysis: (1) Initial Preparation: For impression compound trimming group, timing commenced upon selecting a stock tray and loading it with softened impression compound. It concluded when the preliminary impression trimming was completed. For the light-cured resin group, timing began upon trimming the preliminary impression with a wax knife to uniformly reduce tissue surface thickness by 2 mm. For the CAD/3DP group, timing began with scanning of the preliminary impression using and ended when the STL file was exported. (2) Core Fabrication: For impression compound trimming group, timing covered pouring the preliminary cast with dental stone and minor border adjustments using a laboratory bur. For the light-cured resin group, timing covered resin application onto the preliminary cast, light curing, and mechanical trimming to final dimensions. For the CAD/3DP group, timing included CAD design with mucosal fold extraction, virtual undercut elimination, and 2 mm uniform space creation, followed by slicing in MakerBot Print software. (3) Post-Processing: For impression compound trimming group, timing involved border moulding using occlusion wax and polyether impression recording, including chairside adjustments during functional movements. For the light-cured resin group, timing started at border moulding using occlusion wax and final adjustments. For the CAD/3DP group, timing involved post-printing support removal, edge contouring, and surface polishing.

### 2.5 Dislocation test

Quantitative assessment of tray retention was performed using a universal testing machine (Instron 5,966, United States) under simulated intraoral conditions (37°C, 100% humidity). Custom trays fabricated via three methods were secured to a standardized edentulous mandibular model (Nissan ST-0, Nissin, Japan) using cyanoacrylate adhesive. A 5-mm diameter hemispherical indenter applied vertical dislodgement forces at 1 mm/min crosshead speed until tray separation occurred. Three critical parameters were recorded: Peak dislodgement force (N): Maximum resistance force during separation; Displacement at failure (mm): Tray movement distance before complete dislodgement; Energy absorption (mJ): Area under force-displacement curve.

### 2.6 Statistical analysis

Statistical analyses were performed using SPSS 19.0 (SPSS Inc., United States). Measurable data with normal distribution was described as mean and standard deviations (SD). Block design analysis-of-variance (ANOVA) was used for the analysis of satisfaction levels with the three fabrication methods, while Tukey’s HSD-tests were used for *post hoc* test. A *P*-value less than 0.05 was considered statistically significant.

## 3 Results

The results of the VAS scores for the doctors’ satisfaction levels across six evaluation questions (Q1-Q6) were shown in Table 2. Evaluations of pre-trial tray characteristics (Q1: dimensional accuracy/handling) and border adaptation (Q2: vestibular extensions/frenal clearance) showed pronounced differences among three groups. Operational stability during functional molding (Q3) emerged as the sole parameter without significant variation across techniques (P = 0.839), suggesting this aspect may be operator-dependent rather than methodology-driven. Both impression quality (Q4) and procedural efficiency (Q5) showed statistically significant performance improvements that aligned with the technical complexity of the fabrication methods. These combined improvements resulted in the CAD/3DP approach receiving the highest global satisfaction scores (Q6), with statistically significant advantages over conventional methods (all *P* < 0.001).

**TABLE 2:** VAS score for the doctor’s satisfaction levels with the three tray fabrication methods.

Satisfaction (VAS)	Impression compound trimming	Light curing	CAD and 3DP	F	*P*-value
Q1	57.89 ± 8.27	73.85 ± 6.64^*^	84.97 ± 5.12^*#^	0.169	<0.001
Q2	58.96 ± 9.14	75.92 ± 6.21^*^	86.03 ± 4.83^*#^	0.251	<0.001
Q3	78.43 ± 8.95	80.17 ± 5.72^*^	79.82 ± 4.91^*#^	1.005	0.839
Q4	59.68 ± 8.47	73.52 ± 6.46^*^	86.49 ± 6.35^*#^	0.453	<0.001
Q5	60.21 ± 7.88	73.79 ± 5.95^*^	85.38 ± 6.62^*#^	0.521	<0.001
Q6	59.74 ± 7.83	75.12 ± 6.09^*^	86.55 ± 5.73^*#^	0.437	<0.001

Note: All data are expressed as means ± standard deviations. CAD, computer-aided design; 3DP, three-dimensional printing; Q, question. Block *P*-value indicated the block effect; Methods *P*-value indicated the main effect. * Compared to Impression compound trimming group, *P* < 0.05. # Compared to Light curing group, *P* < 0.05.

The results of Table 3 demonstrated the time required for each manual step and the total manual fabrication time among three groups. Impression compound trimming exhibited the longest total manual duration, particularly during core fabrication phases where it significantly outperformed both light-cured resin and CAD/3DP workflows in time expenditure (*P* < 0.001). CAD/3DP maintained consistent phase-specific efficiency advantages, showing statistically superior performance in post-processing compared to conventional methods (*P* < 0.001). Light-cured resin occupied an intermediate position, with initial preparation times significantly exceeding those of compound trimming (*P* < 0.001) while remaining less efficient than CAD/3DP in critical fabrication stages.

**TABLE 3:** Time required for each manual step and the total manual fabrication time for three methods.

Procedure	Impression compound trimming (s)	Light curing (s)	CAD and 3DP (s)	*F*	*P-value*
Initial Preparation	307.28 ± 12.15	482.36 ± 34.21^*^	427.63 ± 10.88^*#^	168.52	*<0.001*
Core Fabrication	1795.34 ± 76.42	634.19 ± 45.83^*^	763.19 ± 32.74^*#^	432.17	*<0.001*
Post-Processing	496.85 ± 27.15	589.47 ± 38.62^*^	199.83 ± 12.25^*#^	245.89	*<0.001*
Total manual fabrication time	2,578.92 ± 113.45	1705.92 ± 118.76^*^	1,403.74 ± 81.63^*#^	385.74	*<0.001*

Note: All data are expressed as means ± standard deviations. CAD, computer-aided design; 3DP, three-dimensional printing. * Compared to Impression compound trimming group, *P* < 0.05. # Compared to Light curing group, *P* < 0.05.

Dislocation test confirmed significant method-dependent variations across all parameters (*P* < 0.05). The CAD/3DP trays showed significantly better retention and stability than conventional methods (*P* < 0.001), which matched clinicians’ high ratings for edge fit (Q2) and workflow efficiency (Q5). Notably, clinicians reported comparable operational stability across all methods (Q3, *P* = 0.839), suggesting this parameter depends more on operator skill than technical differences (Table 4).

**TABLE 4:** Dislocation results across three methods.

Procedure	Impression compound trimming (s)	Light curing (s)	CAD and 3DP (s)	*F*	*P-value*
Initial Preparation	14.23 ± 1.82	19.58 ± 2.13^*^	25.74 ± 2.63^*#^	86.32	<0.001
Core Fabrication	3.76 ± 0.53	2.89 ± 0.42*	2.14 ± 0.31^*#^	64.15	<0.001
Post-Processing	38.35 ± 5.17	43.82 ± 4.79*	52.06 ± 6.11^*#^	18.97	0.002

Note: All data are expressed as means ± standard deviations. CAD, computer-aided design; 3DP, three-dimensional printing. * Compared to Impression compound trimming group, *P* < 0.05. # Compared to Light curing group, *P* < 0.05.

## 4 Discussion

This study evaluated the clinical usefulness of mandibular edentulous trays fabricated using the CAD and 3DP technique, in comparison with that of trays fabricated using conventional techniques, on the basis of doctors’ satisfaction levels assessed using the visual VAS. Our results suggested that the CAD and 3DP technique is more efficient than conventional techniques for the fabrication of custom edentulous trays, with the satisfaction levels of doctors being higher. Studies showed that mandibular denture stability is clinically the most important factor that influences patient satisfaction (Gonçalves et al., 2022; Alfadda, 2014). Accordingly, we selected 32 patients with a Class III edentulous mandible to ensure standardization and homogeneity in the experiment and ensure that our experimental results reflected actual doctors’ satisfaction. Our findings aligned with recent literature on digital technologies in prosthodontics. Several studies have demonstrated comparable advantages of CAD/3DP custom trays in accuracy and efficiency. For instance, studies reported that digital workflows significantly reduced fabrication time while maintaining or improving precision in removable prosthetics (Tavakolizadeh et al., 2020; Fiorillo et al., 2023). Moreover, studies found that 3D-printed custom trays exhibited superior dimensional stability compared to conventional methods (Wang and Su, 2021; Schmidt et al., 2021). These findings collectively support our observation that CAD/3DP techniques represent a significant advancement in prosthodontic practice.

Impression materials may impact the accuracy of impressions. Lim et al. (2021) compared the accuracy of digital and conventional impression techniques using different dental restorative materials and found no significant difference in accuracy between zirconia or polymethyl methacrylate with conventional impressions and digital impressions. However, Baldissara et al. (2021) reported that new polyvinyl siloxane materials exhibited higher accuracy compared to traditional polyether materials. In recent years, polyether materials have been widely used in dental impressions due to their excellent dimensional accuracy, stability, ease of handling, and superior detail reproduction capabilities (Singer et al., 2023; Zenthöfer et al., 2020). To eliminate discrepancies among different impression materials, we used only polyether to record the final impressions in our study. This study also aimed to identify the relative strengths and weaknesses of the three fabrication methods; therefore, to avoid subjectivity among doctors, three doctors with similar professional titles who had worked in the field of clinical prosthodontics for a similar number of years and had no experience with the CAD and 3DP technique for tray fabrication were specifically selected for a more realistic clinical evaluation. We used the VAS to assess the doctors’ satisfaction levels in this study. Because psychological factors play a role in VAS ratings, evaluations were sequentially conducted for the impression compound trimming technique, CAD and 3DP technique, and light-cured resin technique at 1-week intervals, which served to eradicate the psychological impact of the previous evaluation and yield more representative results.

VAS scores for the doctors’ satisfaction levels showed significant differences (P < 0.001) for the following items: before intraoral trial of the tray (overall size, shape, handling, *etc.*) and border positions during the trial (border extensions, frenal notches); pairwise comparisons also showed significant differences (P < 0.001). These findings indicate that an custom tray fabricated using the CAD and 3DP technique, where the mandibular mucosal fold lines were extracted as margins that were set as borders that intercepted with point cloud data on the preliminary impression of the edentulous maxillofacial tissue surface with the redundant data eliminated, followed by the design of the three-dimensional shape of the handle before fusion of the virtual and actual tray boundaries, created a product with very clear and accurate overall size, shape, border extensions, and frenal notches. Although some minor adjustments were still required during intraoral trials, the doctors were very satisfied with the overall performance of these trays.

With regard to satisfaction with the ease of maintaining tray stability during functional moulding operations, there were no significant differences (*P* > 0.05) among the three techniques. This indicated that the trays fabricated using all three methods can remain stable during functional moulding operations, which is useful for obtaining an accurate final impression. However, it does not mean that a stable tray can definitely achieve an accurate final impression, because accurate positioning of the custom tray in the correct intraoral position is particularly important and requires experience (Schimmel et al., 2023). Our results also demonstrated significant differences in VAS scores for difficulties in achieving a good-quality final impression (repetitions, efficiency) among the three techniques (P < 0.001), with significant differences in pairwise comparisons as well (P < 0.001). These findings also indicate that the main reason why the final impression step needs to be repeated is inaccurate tray positioning (Schimmel et al., 2023). This is evidently a major challenge for inexperienced clinicians. In the CAD and 3DP technique, the vertical, anterior-posterior, and left-right planar dimensions of the primary and secondary denture-bearing areas contain small hemispheres with a radius of 1.5 mm, such that these protrusions separately coincide with the respective insertion directions when the tray is positioned in the oral cavity. This will ensure accurate placement, and if the tray is displaced, the patient will experience pain and discomfort due to the hemispheres. This feature of enhanced stability not only minimizes the difficulties experienced by inexperienced clinicians during functional moulding operations but also decreases the overall chair-side time for both patients and dentists. The dislocation results further verified the advantages of the CAD and 3DP method in terms of tray stability. The peak dislodgement forces and energy absorption values were significantly higher for the CAD/3DP trays compared to the other methods, indicating better retention and stability. These results align with the clinicians’ higher satisfaction with the recorded final impressions, where the CAD/3DP method received the highest satisfaction scores across most criteria. The CAD/3DP trays demonstrated superior border morphology and consistency in impression material thickness, contributing to their higher effectiveness in clinical practice.

With regard to the doctors’ satisfaction levels with the recorded final impressions (completeness, border shape, thickness of impression material), there were significant differences among techniques (P < 0.001), even after pairwise comparisons (P < 0.001). Previous studies have shown that the final impression material must be placed with a uniform thickness on the custom tray (Nanda et al., 2024). The impression compound trimming technique requires that the tissue surfaces and edges of the preliminary impression be trimmed evenly by 2 mm, but in clinical practice, because the lower jaw contains several tissues, it is difficult to evenly trim the paste by 2 mm. Therefore, it is challenging to ensure a final impression that is complete with correct border shapes and uniform thickness. Consequently, doctors were comparatively more satisfied with the light-cured resin and CAD and 3DP techniques in this study. This study is limited by the lack of direct comparisons of the thickness of the final impression material among the three custom trays. However, in the CAD and 3DP technique, the curved surface border of the custom tray is maintained and evenly enlarged along the normal by 2 mm to form the inner surface of the tray, thus reserving a consistent and well-distributed 3D space for the impression material. Previous study (Sun et al., 2019) has shown no differences between the light-cured resin and CAD and 3DP techniques with regard to the thickness of the impression material. In our study, because the time required for the impression compound trimming technique was also included in the time required for the light-curing procedure, we only recorded and compared, in seconds, the manual fabrication time for the light-cured resin and CAD and 3DP techniques, which enabled us to determine a highly effective and satisfying custom tray fabrication technique that clinically saves time and effort. The second limitation of this study was that it eliminated the time required for cast preparation procedures, such as the two-step impression technique for cast fabrication, cast trimming, and base application. The overall tray fabrication time will significantly decrease if these intermediate steps, which are a part of the conventional workflow, are eliminated. Additional limitations included our relatively modest sample size and focus only on Class III ridges, which may limit generalizability to patients with different anatomical presentations. The learning curve for digital workflows, while not specifically measured, could impact clinical adoption. However, the main purpose of the study was to examine the amount of time and effort invested by the doctors, therefore, the time taken for these technical procedures need not be considered. For the CAD and 3DP technique, the impression scanning duration and the time taken to design and polish the custom tray were recorded; the printing time was not considered because the doctors did not participate in this process. For the light-cured resin technique, the time required for impression compound trimming, fabrication of the light-cured custom tray and border moulding was recorded. According to the results, the time taken for impression compound trimming in the light-cured resin technique was significantly lesser than that required for impression scanning in the CAD and 3DP technique (P < 0.001). However, the overall time and the time taken for the remaining steps were significantly lesser for the CAD and 3DP technique than for the light-cured technique (P < 0.001), indicating higher efficiency for the former than for the latter. Therefore, the CAD and 3DP technique can enhance the clinical efficiency of doctors, and its use should be encouraged.

Compared with the time required for border moulding in the light-cured resin technique, that required for contouring in the CAD and 3DP technique was lesser, which decreases the duration of the appointment and enhances patient satisfaction (Prpić et al., 2020). A notable strength of our approach is the combination of subjective evaluations with objective measurements, including the novel dislocation test that provides quantitative evidence supporting the superior retention of digitally designed trays. Therefore, the CAD and 3DP technique can enhance the clinical efficiency of doctors, and its use should be encouraged. Otherwise, we recommend the CAD and 3DP or light-cured resin techniques for custom tray fabrication.

## 5 Conclusion

This study highlights the advantages of utilizing CAD and 3D printing technologies in the fabrication of custom trays for completely edentulous mandibles dentures. Compared to traditional methods, the proposed digital approach demonstrates superior accuracy, efficiency, and user satisfaction among prosthodontists. The findings emphasize the potential of integrating digital design and additive manufacturing techniques to streamline the denture fabrication process, reducing labor intensity while maintaining or enhancing the quality of impressions.

## Data Availability

The original contributions presented in the study are included in the article/supplementary material, further inquiries can be directed to the corresponding author.
